# Fragmentary Provings

**Published:** 1889-06

**Authors:** R. M. Theobald

**Affiliations:** London


### FRAGMENTARY PROVINGS.

R. M. THEOBALD, M. D., LONDON.

*Cubebs*$^{20}$, one dose, produced in a lady patient numbness and coldness, all down right leg from hip to ankle when lying in bed at night; the coldness was objective and subjective.

*Argentum nitricum*$^{200}$ produced in the same patient throbbing headache in vertex, and shooting across vertex.

*Sulphur*$^{900}$, one dose, produced in the same patient dry cough, with pain in left lower chest like a stitch, with tenderness; worse when lying, especially on right side. This improved, but she then had constant headache shooting downward in vertex, aggravated by stooping or looking fixedly, and worse immediately after dinner. Shooting across forehead; eyeballs tender, painful when moved; compelled to close eyes, which relieves the headache. With these symptoms, improvement in the general health, especially a chronic diarrhœa.

